# Soil‐Transmitted Helminthes (STHs) and Asymptomatic Bacteriuria Amongst Preschool Children in the Bosomtwe District of Ghana

**DOI:** 10.1155/bmri/9946374

**Published:** 2026-01-08

**Authors:** Kofi Agyapong Addo, Kwadwo Boampong, Samuel Ayetibo Ofori, Papa Kofi Amissah-Reynolds, Victor Agyei, John Asiedu Larbi

**Affiliations:** ^1^ Department of Biological Sciences Education, AAMUSTED-M, Asante Mampong, Ghana; ^2^ Department of Theoretical and Applied Biology, KNUST, Kumasi, Ghana, knust.edu.gh

**Keywords:** Bosomtwe, minimum inhibitory concentration, preschool children, soil-transmitted helminths, urinary tract infections

## Abstract

Soil‐transmitted helminths (STHs) and urinary tract infections (UTIs) pose major public health challenges, especially in regions with poor healthcare access, inadequate sanitation, and limited clean water supply. When these two conditions recur, they can cause stunted growth in children between 24 and 59 months of age, a vital phase for physical development. This study sought to assess the prevalence of STHs and asymptomatic bacteriuria among preschool children aged 1–5 years in the Bosomtwe District. A total of 344 children from 5 educational circuits were randomly selected for this study. Fecal specimens were obtained from each child and examined for STHs via the formol‐ether concentration method, while urine samples were inoculated onto CLED agar to isolate and identify asymptomatic bacteriuria isolates. Antibiotic susceptibility was assessed using the Kirby–Bauer disk diffusion test. Participant demographics were obtained using a predesigned and structured survey questionnaire. The study found a prevalence of 19.8% (68/344) for STHs and 44.8% (154/344) for bacteriuria, with 6.1% (21/344) having both infections. *Ascaris lumbricoides* was the most common STH, accounting for 12.2% (42/344), followed by *Trichuris trichiura* 4.4% (15/344) and hookworm 3.2% (11/344). More males, 24.2% (40/165), than females, 15.6% (28/179), were affected by STHs. The Kuntenase circuit recorded the highest STH positivity with 26.1% (18/69). For bacteriuria, more females, 47.5% (85/179), than males, 41.8% (69/165) were affected, with age 5 being the most at‐risk group, 46.6% (41/88). *Escherichia coli* was the most isolated Bacteriuria pathogen, 37.5% (129/344), followed by *Klebsiella* spp. 6.1% (21/344) and *Proteus* spp. 1.2% (4/344). Gentamicin was the most effective antibiotic against Bacteriuria isolates. Given the high prevalence of both STHs and bacteriuria, stakeholders should ensure better sanitation and health services, distribute anthelminthic drugs regularly, and raise awareness of bacteriuria in the district.

## 1. Introduction

Soil‐transmitted helminths (STHs) and urinary tract infections (UTIs) pose significant health concerns, particularly in communities where healthcare services, access to safe drinking water, and adequate hygiene infrastructure are lacking [1, 2]. The primary causes of STH infections are four types of worms: roundworms (*Ascaris lumbricoides*), whipworms (*Trichuris trichiura*), and hookworms (*Ancylostoma duodenale* and *Necator americanus*) [3]. These intestinal worms are estimated to infect nearly 2 billion individuals globally, with regions such as the Americas, sub‐Saharan Africa, China, and East Asia experiencing the highest disease burden [4]. Children in preschool and primary school ages are particularly susceptible to STH infections, carrying a significant load of enteric worms compared to other age groups. Consequently, they experience stunted growth, reduced physical fitness, and diminished memory and cognition [5].

Conversely, UTI ranks among the most prevalent bacterial diseases in minors [6], affecting about 150 million people annually and costing the global economy approximately 6 billion US dollars [7]. Most urinary tract infections are caused by bacterial species such as *Escherichia coli*, *Klebsiella*, *Proteus*, *Serratia*, *Enterobacter*, and *Pseudomonas* [8]. In children, *Escherichia coli* is the leading pathogen behind urinary tract infections, accounting for roughly 65%–90% of reported cases [9]. A growing number of bacteria responsible for UTIs are increasingly resistant to commonly prescribed antibiotics, thereby diminishing the effectiveness of managing pediatric UTIs [10].

Existing literature indicates that both STHs and UTIs are prevalent in areas with poor sanitation and unhygienic practices, leading to high morbidity and mortality rates, as well as poor health outcomes [11]. Coinfection of STHs and UTIs was linked to a higher likelihood of developing anemia among school‐aged children [12]. According to Mwang’onde and Mchami, the occurrence of UTIs in Africa varies across different countries and geographic areas. In Ghana, the prevalence is reported at 15.9%, whereas in Senegal, it stands at 4.5%, and in Nigeria, it is 12.3% [13]. Different prevalences of intestinal parasitic infections have been documented in Ghana, with prevalence ranging from 12.1% to 82.9% [14–16]. The occurrence of STH is also common among school children in Ghana. A study conducted among children from ages 2–8 years in Accra of Ghana revealed that 10% were infected with *Giardia lamblia*. Furthermore, another study reported an overall infection rate of 17.3% among children aged 0–10 years, with hookworm as the most prevalent species, followed by *Ascaris lumbricoides* [17].

Further investigation is required to deepen our knowledge of the epidemiology and clinical impacts of coinfections and to guide the development of effective prevention and control measures. Hence, we conducted this study to provide epidemiological data on the coinfection of STHs and asymptomatic bacteriuria in the Bosomtwe district of Ghana.

## 2. Materials and Methods

### 2.1. Study Area

**Figure 1:**
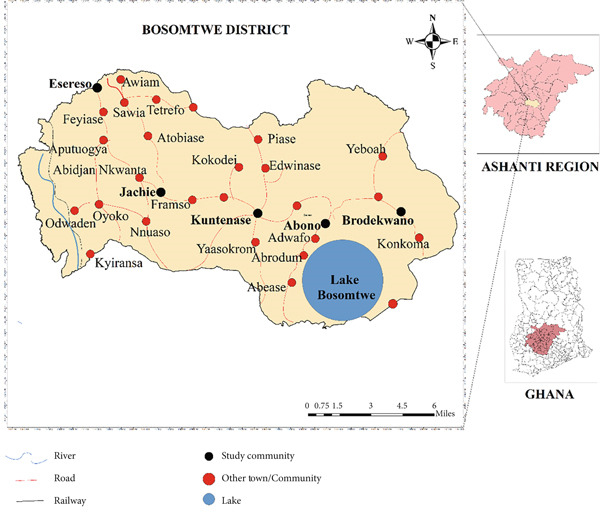
Map of Bosomtwe District. Credit: Map data 2024, adopted from Ghana Statistical Service [18].

The study was carried out in the Bosomtwe District, located in Ghana’s Ashanti Region (Figure 1). Situated about 30 km from Kumasi, the regional capital, Bosomtwe lies between longitudes 0.15°W and 2.25°W and latitudes 5.50°N and 7.46°N, covering a total land area of 24,389 km^2^. The district experiences a rainfall pattern characteristic of the country’s moist semi‐deciduous forest zone, with an average annual temperature of approximately 24°C. The inhabitants are mostly animal or crop farmers or both. Generally, the district has a soil type that has developed over a wide range of weathered rocks. The study area benefits from the Community Water and Sanitation Programme, which supports local initiatives to improve access to clean water and sanitation facilities. Additionally, some households still rely on traditional pit latrines, which are scattered across various communities [18]. In terms of source of drinking water, the communities rely on Lake Bosomtwe (for those at Abono Circuit), boreholes (produced by NGOs), wells, rainwater, and community standpipes. The district was chosen for the study because of multiple pollution challenges arising from domestic activities, industrial operations, and agricultural practices. Additionally, ineffective waste disposal systems and insufficient public facilities pose major challenges in the district [19]. There are five educational circuits in the district under the supervision of the District Education Directorate, Bosomtwe. These are Jachie, Esereso, Abono, Kuntenase, and Brodekwano. Generally, the district consists of towns and villages scattered around the Bosomtwe Lake.

### 2.2. Study Design and Sample Size

A total of 344 preschool children aged 1–5 years were sampled from five circuits (Jachie, Esereso, Abono, Kuntenase, and Brodekwano) in the district. A sample size was determined using the Epi Info calculator, based on an earlier reported prevalence of 15.8% [20], a 95% confidence interval, and 5% error margin. A cross‐sectional survey was conducted across 21 schools, organized under five circuits, to determine the occurrence of STH infections and asymptomatic bacteriuria at individual, circuit, and school levels. Also, factors such as school environmental conditions and the utilization of Lake Bosomtwe were examined as potential risk factors for exposing preschool children to STHs and bacteriuria. The selection of the 21 schools (from five circuits) was purposive, while preschool children meeting the inclusion criteria were chosen randomly.

Eligible participants included preschoolers between the ages of 1 and 5 years residing within the district and whose parents provided assent for their participation in the study. Children who were undergoing medical treatment—such as antibiotics or dewormers for bacteriuria and STHs—were excluded from the study. Exclusion also applied to cases where headteachers were unwilling to release preschool children during the initial or follow‐up visits.

### 2.3. Sample Collection

After meeting with the stakeholders, structured questionnaires were used to collect demographic details and possible risk factors. Each parent/guardian was given a clean container to provide about 3 g of stool and 10 mL of urine from their wards. Parents/guardians were trained to assist children in providing stool samples and clean‐catch midstream urine to minimize contamination. Sample collection was carried out at home in the early morning under parental supervision. The samples were picked up daily at the schools between 6:00 and 6:30 AM, placed in cool boxes containing ice packs, and transferred to the laboratory for same‐day examination to maintain freshness and reliability.

### 2.4. Ethics Statement

Ethical clearance (CHRPE/AP/245/16) was granted by the Committee on Human Research, Publications, and Ethics of Kwame Nkrumah University of Science and Technology (KNUST) and the Komfo Anokye Teaching Hospital. Preschool children obtained parental consent to be included in the study. Approval was also sought from the Bosomtwe District Education Office and Health Directorate. These approvals were communicated to the various headteachers.

### 2.5. STHs Identification

#### 2.5.1. Formo‐Ether Sedimentation Technique

The formo‐ether sedimentation method was employed as described by Uga et al. [21]. This technique involved examining feces for worm eggs and larvae by filtering them through three layers of gauze and washing the resulting material. This method was employed over the Kato–Katz technique, the standard for estimating worm burden, because in several cases, stool samples were preserved at 4°C for 1–2 days to allow time for urinalysis and culture. A 1.5 g sample of feces was utilized, and the pH was adjusted to 3.0 during the process. Wet‐mount microscopy was used to estimate the presence of worm eggs and larvae. Three worms were targeted (hookworms, roundworms, and whipworms) as they are the most frequently observed species in Ghana [22].

### 2.6. Asymptomatic Bacteriuria Identification

#### 2.6.1. Culturing and Identification of Bacterial Isolates

Both cystine lactose electrolyte deficient (CLED) and nutrient agar were obtained (Millipore, Merck) and utilized for the isolation and growth of organisms, respectively. The media preparation strictly followed the manufacturer’s protocol. The serial dilution method was employed for culturing the urine samples. Urine specimens were aseptically cultured on CLED agar plates and maintained at 37°C for a 24‐h incubation period. A bacterial count of ≥ 1 × 10^5^ viable units per milliliter of the sample was considered indicative of clinically significant bacteriuria. Only cultures with one or two bacterial species were considered. Samples with three or more bacterial species were considered contaminated. Contaminated samples were excluded from further analysis, but were, however, collected on second occasions for analysis [23]. Bacterial species were characterized through a combination of microscopic examination (Gram staining) and biochemical tests (catalase, oxidase, indole, coagulase, triple sugar iron [TSI], and citrate tests). Collectively, these assays served as confirmatory tests, aiding in the accurate identification of the bacterial isolates [24].

### 2.7. Antimicrobial Susceptibility Pattern of Isolated Bacteria

The antibiotic sensitivity of the urine isolates to different drugs was assessed using the standard disk‐based antimicrobial testing method. Typically, each circular disk contained 6‐mm filter paper impregnated with known concentrations of antimicrobial compounds. A total of eight antimicrobial agents were evaluated in the study: ciprofloxacin, ceftriaxone, cefotaxime, gentamicin, nalidixic acid, nitrofurantoin, piperacillin/tazobactam, and tetracycline. The bacteria’s susceptibility to the antibiotic was subsequently assessed by measuring the radius of the inhibition zone. This was done by starting from the center of the disk and comparing the measurement with a set of standardized values. These reference values differ based on the specific antibiotic used and its dosage concentration.

### 2.8. Statistical Analyses

Data collected from participants were first organized in Microsoft Excel and subsequently transferred to SPSS software version 15 for statistical evaluation. The prevalence and *t* tests for STHs and UTIs and their coinfection were computed using descriptive statistics. Statistical tools such as the *χ* ^2^ test and odds ratio (OR) were applied to examine the association between infection rates and environmental determinants influencing disease outcomes.

## 3. Results

### 3.1. Participants’ Demographic Characteristics

A total of 344 preschoolers (between 1 and 5 years) participated in the study. Among them, 165 (48.0%) were female and 179 (52.0%) were male. Children aged 4 years formed the largest group, accounting for 37.5% (129/344) of the sample, followed by those aged 3 years at 32.0% (110/344). Participants aged 5 and 2 years comprised 25.6% (88/344) and 4.7% (16/344), respectively. The average age of the children was 3.83 years, with a standard deviation of 0.87.

### 3.2. Prevalence of STH From Stool Samples

An overall prevalence of 19.8% (68/344) was recorded among the preschool children sampled for this study. As indicated in Table 1, STH infection was observed in 24.2% (40/165) male pupils and 15.6% (28/179) female pupils; however, this variation did not reach statistical significance (*p* = 0.062). Children aged 5 years recorded the highest prevalence at 23.9% (21/88), while children aged 2 years had the lowest prevalence at 6.3% (1/16). The prevalence showed an upward trend with increasing age; however, the relationship did not demonstrate statistical significance (*p* = 0.388). Participants from the Kuntenase circuit recorded the highest STH prevalence of 26.1% (18/69), followed by Brodekwano 24.2% (16/66) and Esereso 24.2% (8/33). The prevalence was comparatively lower in the Abono (14.5%; 16/110) and Jachie (15.2%; 10/66) circuits. Nonetheless, the variation in STH‐positive cases across the circuits did not present a statistically meaningful difference (*p* = 0.217). Of the helminth species detected, *Ascaris lumbricoides* occurred most frequently, infecting 12.2% (42/344) of the participants, while *Trichuris trichiura* and hookworm were identified in 4.4% (15/344) and 3.2% (11/344) of the children, respectively. The frequencies of the worms identified differed significantly (*p* = 0.001).

**Table 1:** Prevalence of STH among study participants.

**Variables**	**Category**	**Number of participants (** **n** = 344**)**	**Positive % (*n*)**	**Negative % (*n*)**	***χ*** ^**2**^ **-value**	***p* value**
Gender	Male	165	24.2 (40)	75.8 (125)	3.479	0.062
	Female	179	15.6 (28)	84.4 (151)		

Age (years)	1	1	0 (0)	100 (1)	4.129	0.388
	2	16	6.3 (1)	93.7 (15)		
	3	110	16.4 (18)	83.6 (92)		
	4	129	21.7 (28)	78.3 (121)		
	5	88	23.9 (21)	76.1(67)		

Circuits	Jachie	66	15.2 (10)	84.8 (56)	5.765	0.217
	Esereso	33	24.2 (8)	75.8 (25)		
	Abono	110	14.5 (16)	85.5 (94)		
	Kuntenase	69	26.1 (18)	73.9 (51)		
	Brodekwano	66	24.2 (16)	75.8 (50)		

Identified Worms	*Ascaris lumbricoides*	344	12.2(42)	87.8 (302)	26.858	<0.001
	*Trichuris trichiura*	344	4.4 (15)	95.6 (329)		
	Hookworm	344	3.2 (11)	96.8 (333)		

### 3.3. Prevalence of Bacteria Isolated From Urine Samples

An overall prevalence of 44.8% (154/344) was recorded for asymptomatic bacteriuria among the preschool children. More females (47.5%, 85/179) tested positive for asymptomatic bacteriuria than males (41.8%, 69/165). The prevalence rates were fairly similar across the age groups, with 5‐year‐olds exhibiting the greatest rate at 46.6% (41/88). Across the educational circuits, children in the Kuntenase circuit recorded the highest prevalence of asymptomatic bacteriuria at 55.1% (38/69), followed by Abono at 44.5% (49/110), Brodekwano at 42.2% (28/66), and Jachie and Esereso, each recording 39.4% (26/66 and 13/33, respectively). *Escherichia coli* emerged as the predominant uropathogen isolated, representing 37.5% (129/344) of the total asymptomatic bacteriuria detected. This was followed by *Klebsiella* spp. 6.1% (21/344), and *Proteus* spp. was the least isolated organism at 1.2% (4/344), as shown in Table 2.

**Table 2:** Prevalence of asymptomatic bacteriuria among study participants.

**Variables**	**Category**	**Number of participants (** **n** = 344**)**	**Positive % (*n*)**	**Negative % (*n*)**	***χ*** ^**2**^ **-value**	***p* value**
Gender	Male	165	41.8 (69)	58.2 (96)	0.89802	0.3433
	Female	179	47.5 (85)	52.5 (94)		

Age (years)	1	1	0 (0)	1 (100)	1.0526	0.9017
	2	16	43.8 (7)	56.2 (9)		
	3	110	45.5 (50)	54.5 (60)		
	4	129	43.4 (56)	56.6 (73)		
	5	88	46.6 (41)	53.4 (47)		

Circuits	Jachie	66	39.4 (26)	60.6 (40)	4.2682	0.3709
	Esereso	33	39.4 (13)	60.6 (20)		
	Abono	110	44.5 (49)	55.5 (61)		
	Kuntenase	69	55.1 (38)	44.9 (31)		
	Brodekwano	66	42.2 (28)	57.8 (38)		

Isolated bacteria	*Escherichia coli*	344	37.5 (129)	62.5 (215)	210.49	< 0.001
	*Klebsiella* sp.	344	6.1 (21)	93.9 (323)		
	*Proteus* spp.	344	1.2 (4)	98.8 (340)		

### 3.4. Association of Intestinal Helminths by Sex, Age, and Location

*Ascaris* represented the predominant helminthic infestation (42 cases, 12.2%), with *Trichuris* (15 cases, 4.4%) and hookworm (11 cases, 3.2%) occurring less frequently. Among female participants (*n* = 179), a statistically significant relationship was observed between parasite presence and helminth species (*p* = 0.002), while no such relationship was detected among males (*n* = 165). Infections were more frequent in age 4, where the prevalence of *Ascaris* reached 18 (23.5%). Circuit analysis indicated higher infection rates in Kuntenase (12 *Ascaris*, 5 *Trichuris*, 1 hookworm) and Brodekwano (11, 1, and 4), respectively, with significant clustering observed in Abono (*χ* ^2^ = 8.46, *p* = 0.014) and Kuntenase (*χ* ^2^ = 7.96, *p* = 0.018). These findings suggest that age and locality play important roles in helminth transmission within the study area (Table 3).

**Table 3:** Association of intestinal helminths by sex, age, and location.

**Factors**	***Ascaris n* (%)**	***Trichuris n* (%)**	**Hookworm *n* (%)**	***χ*** ^**2**^	***p* value**
Sex					
Male	24 (35.3%)	9 (13.2%)	7 (10.3%)	0.199	0.655
Female	18 (26.5%)	6 (8.8%)	4 (5.9%)	12.68	0.002
Age					
1	0 (0.0%)	0 (0.0%)	0 (0.0%)		
2	1 (6.2%)	0 (0.0%)	0 (0.0%)	2.043	0.360
3	13 (19.1%)	3 (4.4%)	2 (2.9%)	6.39	0.041
4	16 (23.5%)	7 (10.3%)	5 (7.4%)	10.62	0.005
5	12 (17.6%)	5 (7.4%)	4 (5.9%)	9.16	0.010
Circuit					
Jachie	4 (6.1%)	3 (4.4%)	3 (4.4%)	3.09	0.212
Esereso	5 (15.2%)	2 (6.1%)	1 (3.0%)	3.54	0.170
Abono	10 (9.1%)	4 (5.9%)	2 (1.8%)	8.46	0.014
Kuntenase	12 (17.4%)	5 (7.4%)	1 (1.5%)	7.96	0.018
Brodekwano	11 (16.7%)	1 (1.5%)	4 (6.1%)	4.78	0.091

### 3.5. Antibiotic Susceptibility Pattern of Isolated Bacteria

*E. coli* showed susceptibility to gentamicin (96.1%), nitrofurantoin (93.8), and ciprofloxacin (93.0%) but resistance against cefotaxime (88.3%). On the other hand, *Klebsiella* sp. isolates showed equal susceptibility patterns against nitrofurantoin (90.5%) and ciprofloxacin (90.5%) but resistance against cefotaxime (90.5%). Additionally, the highest susceptibility patterns of *Proteus* spp. were observed for gentamicin (100%), nalidixic acid (100%), nitrofurantoin (100%), and ceftriaxone (100%) but resistance against cefotaxime (100%). Table 4 presents the antimicrobial sensitivity profiles of the bacterial isolates.

**Table 4:** Antibiotic susceptibility and resistance characteristics of isolates.

**Antibiotic**	***Escherichia coli* (** **N** = 129 **)**		***Klebsiella* sp. (** **N** = 21**)**		***Proteus* spp. (** **N** = 4**)**	
	**Susceptibility (%)**	**Resistance (%)**	**Susceptibility (%)**	**Resistance (%)**	**Susceptibility (%)**	**Resistance (%)**
Ciprofloxacin (CIP)	93.0 (120)	22.7 (29)	90.5 (19)	9.5 (2)	75 (3)	25 (1)
Ceftriaxone (CTR)	60.5 (78)	39.6 (51)	4.7 (6)	71.4 (15)	100 (4)	0
Cefotaxime (CTX)	11.6 (15)	88.3 (114)	9.5 (2)	90.5 (19)	0	100 (4)
Gentamicin (GEN)	96.1 (124)	3.9 (5)	81.0 (17)	19.0 (4)	100 (4)	0
Nalidixic acid (NA)	66.7 (86)	33.1 (43)	90.5 (19)	63 (3)	100 (4)	0
Nitrofurantoin (NIT)	93.8 (121)	6.5 (8)	90.5 (19)	9.5 (2)	100 (4)	0
Piperacillin/tazobactam (PIT)	86.0 (111)	13.6 (18)	38.1 (8)	62 (13)	25 (1)	75 (3)
Tetracycline (TET)	63.6 (82)	36.4 (47)	19.4 (4)	81 (17)	25 (1)	75 (3)

### 3.6. Coinfection of STH and Asymptomatic Bacteriuria

Out of the 344 preschool children examined for STHs and asymptomatic bacteriuria, 6.1% (21) were observed to harbor both STH and asymptomatic bacteriuria, with females (3.8%) having a higher prevalence than males (2.3%). Preschoolers aged 4 years showed the greatest rate of coinfection at 7.0%, whereas no cases of concurrent STH and bacteriuria were detected among 1‐year‐olds, as presented in Table 3. Among the Circuits, coinfection of STH and asymptomatic bacteriuria were mostly recorded in Abono (10%), followed by Brodekwano and Jachie with 9.1% and 1.6%, respectively. Esereso recorded no coinfection. The difference in the number of preschool children who were identified with coinfections in different circuits differed statistically (*p* = 0.030), as shown in Table 5.

**Table 5:** Coinfection of STHs and UTIs.

**Variables**	**Category**	**Number of participants (** **n** = 344**)**	**(STH+ bacteriuria)** **Positive (%)**	**Negative (%)**	***χ*** ^**2**^ **value**	***p* value**
Gender	Male	165	2.3 (8)	97.7 (157)	0.503	0.478
	Female	179	3.8 (13)	96.2 (166)		

Age	1	1	0 (0)	1 (100)	0.345	0.986
	2	16	6.3 (1)	93.7 (15)		
	3	110	5.5 (6)	94.5 (104)		
	4	129	7.0 (9)	93.0 (120)		
	5	88	5.7 (5)	94.3(83)		

Circuits	Jachie	66	1.6 (1)	98.4 (65)	10.697	0.030
	Esereso	33	0.0 (0)	100 (33)		
	Abono	110	10.0 (11)	90.0 (99)		
	Kuntenase	69	4.3 (3)	95.7 (66)		
	Brodekwano	66	9.1 (6)	90.9 (60)		

## 4. Discussion

Bacteriuria and soil‐transmitted helminths (STHs) present major health challenges, especially among children aged 1–5 years, thereby raising concerns for public health [2]. STHs are common parasites that cause infections in pediatric populations worldwide. The study found an overall STH infection rate of 19.8% among preschoolers (aged 1–5), highlighting a significant public health concern. These infections may contribute to child illness, growth retardation, reduced physical performance, and cognitive or memory impairments in children within the district [25, 26]. Adu‐Gyasi et al. reported that the prevalence of STH infections varies considerably by location, with these infections being particularly common in tropical and subtropical areas [16]. The overall prevalence observed in this study was notably higher than the 6% reported by Ofosu et al. [27] in the Kwahu West Municipality and the 9.4% documented by Sam [28] in Gia and Kajelo within the Kassena‐Nankana East and West Districts of Ghana’s Upper East Region. This finding aligns with the observation by Prakash et al., who reported that STHs and *S. mansoni* are endemic around Lake Bosomtwe, largely as a result of environmental pollution [29].

The STH prevalence observed in this study is lower than that reported in research from other African nations. For example, Kepha et al. [30] found a 27.8% prevalence in Bumula District, Western Kenya, whereas Ikeoluwapo et al. [31] reported a 35.9% prevalence in Ibadan, Nigeria. Furthermore, Tilahun et al. [32] observed a notably high prevalence of 54.9% among school‐aged children in Durbete Town, northwestern Ethiopia. Such differences in prevalence could be linked to variations in environmental conditions. Soil acts as the main reservoir for helminth eggs, and in areas with poor environmental sanitation, it can become heavily laden with large numbers of these eggs. Individuals may then become infected through ingestion or direct contact with contaminated soil [3].

*Ascaris lumbricoides* (12.1%), *Trichuris trichuria* (4.4%), and hookworm (3.2%) were identified as the main causative agents of soil‐transmitted helminthiasis in the district, although a few cases of *Strongyloides* and *Schistosoma hematobium* were observed but not reported. The average temperature of 24°C in the Bosomtwe District creates favorable conditions for the development of helminth eggs, especially those of *Ascaris* and *Trichuris*. Studies suggest that children harboring *Ascaris lumbricoides* infections have a higher risk of growth stunting compared with their uninfected peers. Additionally, infection with Hookworm is recognized as a major etiological factor contributing to anemia worldwide [5].

In this study, STHs were observed to be more prevalent in males, affecting 11.7% of them, whereas females showed a lower prevalence of 8.1%. While no specific hypotheses have been proposed regarding the relationship between gender and STH infections, it is generally observed that infant males tend to engage in more soil‐related activities than females. The prevalence also increased with age, the use of the lake or other nearby streams and living in uncemented compounds. The distribution and patterns of helminth infections are shaped by multiple determinants, such as the surrounding environment, the heterogeneous characteristics of the population, age, family composition, genetic background, and the presence of coinfections with multiple parasites (polyparasitism) [33]. Interestingly, STHs were found to be more prevalent in areas farther away from the lake. This phenomenon may be attributed to the increased attention given by the health directorate to towns closer to the lake. As a result, these areas have better control measures and lower prevalence rates.

Studies show that children below 14 years are especially susceptible to STH infections because of their developing immune systems and elevated nutritional needs [34]. These factors can impact various aspects of their health, including excretion, metabolism, nutrient intake, and energy levels. In endemic communities, STH infections tend to be aggregated, with most infected individuals experiencing diseases of moderate or light intensity, while a smaller number may be heavily infected. People harboring heavy infections often act as the main source of transmission for the broader community [35]. Preventive chemotherapy (anthelminthic medicines) can be administered once a year to effectively manage and control STH infections in the community [36]. However, barriers to treatment include low caregiver awareness, irregular drug supply, and prevailing myths about deworming in the district, although the cost of albendazole or mebendazole treatment is less than US$0.05 per child per year.

According to Chang and Shortliffe, pediatric UTIs can potentially result in irreversible renal damage and significant acute morbidity if not treated appropriately [37]. However, kidney damage is rare. In most cases, pediatric UTIs result in pain, discomfort, and school absenteeism, which disrupt learning and quality of life. Some children may also present with asymptomatic bacteriuria, whose long‐term effects remain uncertain. The prevalence of asymptomatic bacteriuria reported in this study (44.8%) was higher than the 15.8% reported by Ephraim et al. [20], in a similar study conducted in the Assin‐South Municipality, Ghana. However, the prevalence observed in the current study is nearly comparable to the 44.0% reported by Oluwagunke [38] in Kwara State, Nigeria.

Other regions have reported varying prevalences. Muoneke et al. [39] documented a 3% prevalence among children in Abakaliki, which is markedly lower than the rate observed in the present study. Likewise, Bahati et al. [40] recorded a prevalence of 20.3%, which remains below the rate identified in the present study. Again, another study investigating the prevalence and risk factors for UTIs and severe malaria among febrile children at Makango Health Centre in Mwanza City, northwestern Tanzania, Epaphura et al. [41] documented a prevalence of 39.7%. UTIs are frequently linked to inadequate personal hygiene and suboptimal environmental conditions, including insufficient sanitation facilities, limited access to clean water, and poor waste management systems. The differences in prevalence rates observed in this study compared to others may be due to variations in geographical locations as well as the factors previously discussed. It is essential to emphasize the importance of maintaining cleanliness and proper disposal of human waste to promote good health and prevent UTIs [7, 10]. In this study, females were the most affected by asymptomatic bacteriuria within the district. This observation is consistent with a WHO report indicating that females are generally more prone to UTIs [42]. It is also consistent with findings from studies by Ephraim et al., Oluwaguneke, Muoneke et al., and Bahati et al. [20, 38–40], which have reported similar patterns of UTI distribution by gender.

If the asymptomatic bacteriuria prevalence of 44.8% remains unaddressed, there is a risk it could increase in the coming years. This would leave children vulnerable to serious health issues. These risks include acute illness, pain, discomfort, and recurrent absenteeism, which collectively impair growth and development. This can also lead to the development of hypertension in adulthood [37]. Environmental factors could contribute to the prevalence of UTIs in these regions, emphasizing the need to improve sanitation and water quality to lessen the impact of UTIs on children. However, factors such as the use of the lake or other nearby streams and the nature of participants’ compounds, i.e., whether the compounds are cemented or not, did not significantly affect the prevalence of UTIs.

*Escherichia coli* was the most frequently detected organism in this study, appearing across all circuits and showing the highest occurrence in the Abono circuit. *Klebsiella* spp. were found in just three of the circuits. These results are in agreement with the studies of Bahati et al., Oluwaguneke, and Sunita et al. [38, 40, 43] who also identified *E. coli* as the most commonly recovered organism, reflecting similar trends observed in the present study. Riccabona [44], also noted that gram‐negative bacteria, especially *E. coli*, represent the predominant pathogens recovered from urine samples of children with uncomplicated urinary tract infection.

It is crucial to take into account current antimicrobial resistance trends when choosing an appropriate medication for treating UTIs. In this study, Gentamicin was the most effective antibiotic that inhibited the growth of *Proteus* spp., *E. coli*, and *Klebsiella* spp. isolates. This result in the antimicrobial susceptibility profile is consistent with the findings of Ephraim et al. [20] who identified Gentamicin as the preferred treatment in their study. It also agrees with the drugs suggested by Ghana Standard Treatment Guidelines, which recommends Gentamicin for the treatment of complicated UTI infections [45]. On the contrary, Prah et al. [46] identified Amikacin as the most effective agent against UTI isolates. Additionally, other studies have reported *E. coli* resistance to Ciprofloxacin, which differs from the findings of the present study [47]. The antibiotic sensitivity of UTI isolates varies over time and differs across various regions [48]. The differences observed in susceptibility and resistance patterns may result from mutations in chromosomal genes or the uptake of external genetic resistance elements, frequently sourced from naturally resistant environmental organisms [49].

The occurrence of both STH infections and asymptomatic bacteriuria among children underscores the intricate relationship between parasitic and bacterial pathogens in vulnerable groups, where inadequate hygiene, poor sanitation, and altered host immunity may increase the risk of dual infections. Several studies have reported coinfections, but mostly on malaria‐helminths, urogenital schistosomiasis‐STHs with prevalences of 4.5% and 4.7%, respectively [50, 51]. Coinfections of asymptomatic bacteriuria and STHs are rarely documented. The observed 6.1% coinfection rate of bacteriuria and STHs underscores the urgent requirement for focused public health interventions to mitigate this dual burden. From our checklist, most of the schools had uncemented compounds, poor sanitation facilities, and refuse dump sites located close to the classroom blocks. Such conditions create favorable environments for the proliferation of both bacteria and helminths. In addition, the schools were unfenced, allowing stray animals to enter the compounds and increasing the risk of transmission through their potential role as mechanical vectors. Beyond higher morbidity and increased school absenteeism among school children, managing two concurrent infections can place a significant strain on healthcare resources and adversely affect the overall well‐being of the affected children [52]. Improving sanitation, promoting hygiene, and implementing integrated health programs are crucial steps in reducing the prevalence and impact of coinfections among vulnerable populations, particularly in Abono, which recorded the highest prevalence and continues to serve as a resort center for many visitors to the lake.

## 5. Conclusion

STHs and asymptomatic bacteriuria are still endemic in the Bosomtwe District of Ghana. Poor environmental conditions of the basic schools, such as unhygienic environments and poor dampening settings, are major contributing factors to the disease occurrence. Strengthening of control strategies is required to mitigate the burden of STHs and asymptomatic bacteriuria. This can be done through effective health education campaigns and school screening programs. District health directorate stakeholders should strengthen health education efforts to raise community awareness, which is crucial for reducing helminth infections. These initiatives should be carried out thoroughly to counteract the misconceptions, superstitions, and political influences that the population associates with health matters.

## 6. Limitations of the Study

### 6.1. Symptomatic vs. Asymptomatic Bacteriuria

This study did not include a detailed assessment of clinical symptoms to distinguish between symptomatic and asymptomatic bacteriuria. We acknowledge this as a limitation, as incorporating symptom analysis would have provided a clearer understanding of the clinical relevance of bacteriuria in the study population.

## Conflicts of Interest

The authors declare no conflicts of interest.

## Author Contributions

K.A.A., S.A.O., P.K.A.R., and V.A. were responsible for collecting field data, as well as performing laboratory work and data analysis. K.A.A., K.B., and S.A.O. prepared the manuscript, with J.A.L. supervising the study. All authors participated in refining the manuscript through proofreading, making revisions, and approving the final version for publication.

## Funding

No funding was received for this manuscript.

## Data Availability

The data that support the findings of this study are available from the corresponding author upon reasonable request.
